# Impaired spontaneous belief inference following acquired damage to the left posterior temporoparietal junction

**DOI:** 10.1093/scan/nsw076

**Published:** 2016-06-17

**Authors:** Aurélie Biervoye, Laurence Dricot, Adrian Ivanoiu, Dana Samson

**Affiliations:** ^1^Psychological Sciences Research Institute, Université catholique de Louvain, Louvain-la-Neuve, Belgium; ^2^Institute of Neuroscience, Université catholique de Louvain, Brussels, Belgium,; ^3^Department of Neurology, Saint-Luc University Hospital, Brussels, Belgium

**Keywords:** social cognition, theory of mind, spontaneous belief reasoning, temporoparietal junction

## Abstract

Efficient social interactions require taking into account other people’s mental states such as their beliefs, intentions or emotions. Recent studies have shown that in some social situations at least, we do spontaneously take into account others’ mental states. The extent to which we have dedicated brain areas for such spontaneous perspective taking is however still unclear. Here, we report two brain-damaged patients whose common lesions were almost exclusively in the left posterior temporoparietal junction (TPJp) and who both showed the same striking and distinctive theory of mind (ToM) deficit. More specifically, they had an inability to take into account someone else’s belief unless they were explicitly instructed to tell what that other person thinks or what that person will do. These patients offer a unique insight into the causal link between a specific subregion of the TPJ and a specific cognitive facet of ToM.

## Introduction

Theory of mind (ToM) refers to the cognitive ability to infer and reason about our own and other people’s mental states (beliefs, intentions or emotions; Premack and Woodruff, 1978). Recently, scientists discovered that ToM does not only rely on complex and effortful cognitive processes but that it also relies on processes that are available at a very young age and that can be triggered spontaneously, in certain situations at least (Onishi and Baillargeon, 2005; Kovács *et al.*, 2010; Samson *et al.*, 2010; Schneider *et al.*, 2014a; Hyde *et al.*, 2015). Being able to spontaneously track others’ mental states is thought to be a crucial facet of our social skills and has been proposed to be at the heart of the mentalizing deficits observed in individuals diagnosed with Autism Spectrum Disorder (Senju *et al.*, 2009; Schneider *et al.*, 2013). So far, it is unclear, however, whether there are dedicated brain areas that sustain our ability to spontaneously track others’ mental states.

The different brain areas involved in ToM have been clearly identified and encompass the temporal poles, the temporoparietal junction (TPJ), the lateral prefrontal cortex, the posterior cingulate and the median prefrontal cortex (mPFC) (for a recent meta-analysis see Schurz *et al.*, 2014). So far, it is mainly the overlap between the ToM brain areas involved during on command belief reasoning and those involved in spontaneous belief reasoning that have been highlighted. For example, the right TPJ has been found to be activated not only when healthy participants are directly asked to mentalize but also when mentalizing is required as part of another judgment (such as in the case of moral judgments; e.g. Young and Saxe, 2009) or even simply when participants observe without mentalizing instructions a scenario in which a protagonist holds a false belief (Kovács *et al.*, 2014; Schneider *et al.*, 2014b; Hyde *et al.*, 2015). Overlap between on command and spontaneous belief reasoning has also been observed in the left anterior superior temporal sulcus and precuneus (Schneider *et al.*, 2014b). Such overlap suggests at least partially common processes when mentalizing is initiated by task instructions and when it is initiated spontaneously. Is there however a specific functional and neural mechanism that triggers mentalizing processes in the absence of explicit instructions? The two cases of brain-damaged patients we report here, directly speak to this issue.

The two cases we report here had common brain lesions within the left TPJ and were both impaired in tracking other people’s beliefs spontaneously. To demonstrate the specificity of the patients’ deficits, it was important to show (i) that they were unimpaired when explicit belief reasoning instructions were given to them and (ii) that their difficulties could not be explained by another type of mentalizing deficit. We thus presented four belief reasoning tasks designed to manipulate not only the need for spontaneous belief tracking but also two other types of mentalizing demands that previous brain-damaged patients were shown to be sensitive to. The need for spontaneous belief tracking was manipulated by changing the task instructions. In one condition, participants were asked to identify or localize an object. There were no explicit mentalizing instructions, but inferring one of the protagonist’s beliefs was necessary to solve the task. In the other condition, participants were invited to explicitly pay attention to the protagonist’s actions and thoughts. The need to inhibit irrelevant distractors was a second mentalizing demand that was manipulated. In one condition, salient but misleading distractors were provided in the environment. In the other condition, no distractors were present. This manipulation was included since a previous case of impaired belief reasoning was interpreted as resulting from a selective deficit in resisting interference from salient but irrelevant distractors in the environment (Samson *et al.*, 2004, 2007). Finally, self-perspective inhibition was the third source of mentalizing demands that was manipulated. In one condition, the participants knew the identity or location of the object and had to inhibit this knowledge to infer the protagonist’s belief. In the other condition, participants were unaware of the object’s true identity or location, a situation which diminished the self-perspective inhibition demands. This latter manipulation was included since the ability to inhibit one’s own perspective can be selectively disrupted following brain-damage (Samson *et al.*, 2005, 2015). The combination of mentalizing demands was different in each of the four belief reasoning tasks so that we could identify which manipulation affected the patients’ performance. We predicted that if the deficit of the two patients was selective to conditions where beliefs had to be tracked spontaneously, then their performance should only be affected by the task instruction manipulation and not by the manipulation of demands in terms of distractor inhibition or self-perspective inhibition.

## Methods

### Case description

Patient IM, a 43-year-old French-speaking and right-handed female first consulted a neurologist due to headaches with neck pain. The magnetic resonance imaging (MRI) scan performed in 2005 showed lesions in the left parietal-occipital junction and in the left superior cerebellar presumptively of vascular origin. At the time of testing, patient IM took anticoagulants and medication for arterial hypertension. Patient KV was a 60-year-old female and retired employee at the time of testing. She was bilingual in French and Dutch and a forced right-hander (she wrote with the right hand since childhood). She suffered from a cerebral anoxia in 2007 (cardiac arrest during a thyroidectomy) which affected the left frontal and the bilateral parieto-occipital regions. At the time of testing, she took an antidepressant as well as thyroid and insulin medication. The two patients had no history of drug or nicotine use or alcohol abuse. The two patients had no known common medical history prior to their neurological incident. The neuropsychological profile of both patients is presented in Supplementary Table S1.

During the first clinical interviews after their cerebral damage (more than 5 years prior to our testing), the two patients complained mainly of difficulties in memory and fatigue. Their spontaneous complaints, although still present, were significantly reduced at the time of testing (as corroborated by the improvement in the neuropsychological tests, Supplementary Table S1). They had no spontaneous complaints about their social interactions and they reported healthy relationships with their family, friends, etc. Their global scores at a questionnaire assessing cognitive and affective empathy (French translation of the Questionnaire of Cognitive and Affective Empathy, a QCAE from Reniers *et al.*, 2011) was within the norms both for the cognitive empathy subscale (patient IM = 57; patient KV = 68; controls’ mean = 59.42, s.d. = 7.59) and for the affective empathy subscale (patient IM = 42; patient KV = 39; controls’ mean = 36.76, s.d. = 5.06). Note that these were only self-reported measures (we were not able to collect data from heteroevaluation). When asked more specific questions about their daily life social interactions, both patients reported however more frequent misunderstandings of what other people meant or intended to do since their neurological incident. Such misunderstandings were reported when the meaning or intention was implicit rather than explicit.

### Control participants

Ten healthy participants were recruited as control participants for the behavioral tasks. Five controls were matched to patient IM for age (mean age = 45 years, range = 41–48 years) and education (they had a bachelor’s degree). The other five healthy participants were matched to patient KV for age (mean age = 61 years, range = 57–65 years) and education (basic secondary). Not all these control participants were willing or allowed to undergo MRI examination, which led us to recruit additional healthy participants to reach a total of 18 healthy participants as control for the neuroanatomical analysis. Ten controls were matched to patient IM for age (mean age = 44.2 years, range = 38–49 years), and eight were matched to patient KV for age (mean age = 59 years, range = 56–65 years).

### Neuroanatomical analysis

All MRI data [three-dimensional (3D) anatomical data] were acquired on a 3T scanner with a 32-channelphased-array head coil (Achieva, Philips Healthcare, Eindhoven, The Netherlands). The anatomical 3D sequence consisted of a gradient-echo sequence with an inversion prepulse (Turbo Field Echo) acquired in the sagittal plane, using the following parameters: repetition time = 9.1 ms, echo time = 4.6 ms, flip angle = 8°, number of slices = 150, slices thickness = 1 mm, in-plane resolution = 0.81 × 0.95 mm^2^ (acquisition) reconstructed in 0.75 × 0.75 mm^2^, field of view = 237 × 197 mm^2^, acquisition matrix = 296 × 251 (reconstructed in 320^2^), sensitivity encoding (SENSE) factor = 1.5 (parallel imaging), total scan time = 8 min 50 s per run.

Surface-based analyses were performed using FreeSurfer (FreeSurfer; Martinos Center for Biomedical Imaging, Boston, MA, USA). Both patients were compared with the control group (*n* = 18). The data were corrected for multiple comparisons *q*(false discovery rate, FDR) < 0.05. Moreover, each patient was compared with her matched control group (*n* = 10 for patient IM, *n* = 8 for patient KV). The data were corrected for multiple comparisons *q*(FDR) < 0.05.

### Behavioral tasks

In each false-belief task, the instructions were explained to the participant before starting the task. Participant performed practice trials to check their comprehension of the task and were then presented with the test trials. After each trial, the participant received a feedback consisting of a picture with the correct response. The trials within a task were presented in pseudorandom order (ensuring that they were never more than three trials of the same type one after the other).

#### Task 1: reality unknown false-belief task without distractors

This task consisted of a shortened version of the non-verbal videos used in previous studies (Apperly *et al.*, 2004; Samson *et al.*, 2004). For each video, the participants’ task was to find which of two identically looking boxes contained a green object. Participants were told that the woman in the video would help them find the location of the hidden objet by placing a pink object on the top of one of the two boxes.

The videos started by showing a man who hid a green object in one of two boxes placed in front of him. The man showed to the woman where the green object was located. Although the woman could see the location of the object, the participants could not see the location because of the camera’s angle. In the false-belief trials, the woman then left the room. While she was out, the man swapped the boxes. When she returned, she indicated one of two boxes with the pink object. To find the correct location of the green object, participants had to infer that the woman had a false belief about the green object’s location (because the boxes were swapped during her absence) and they had thus to choose the box opposite to the one the woman pointed at.

These false-belief trials (*n* = 12) were mixed with true-belief trials (*n* = 12), memory control trials (*n* = 12), inhibition control trials (*n* = 6) and fillers trials (*n* = 6). These additional trials were used to check if a deficit in this task was caused by other cognitive difficulties than inferring false beliefs (e.g. inhibition or memory problems; see Samson *et al.*, 2004, for the details about these additional trials). All 48 trials were presented in two different blocks during the same session.

In this version of the false-belief task, the demands in terms of spontaneous belief tracking were high because the test question (‘In which box is the green object located?’) did not explicitly refer to the woman’s mental state. Yet, to successfully locate the green object on false-belief trials, participants had to take the woman’s belief into account. The demands in terms of self-perspective inhibition were low because on false-belief trials, participant had no idea where the green object was located before they inferred that the woman had a false belief. Thus, at the time of belief inference there was no need to resist interference from one’s own knowledge of the true location. And finally, the demands in terms of inhibition of irrelevant distractors were low as no irrelevant information about the object’s location was provided. (Note that the woman’s pointing with the pink marker was relevant to the task and not a distractor.)

#### Task 2: reality known false-belief task without distractor

This task was a shortened version of the task used in a previous study (Samson *et al.*, 2005) and consisted of similar non-verbal videos as task 1. However, there were two important changes. First, at the point when participants could infer the woman’s belief, they knew the new location of the green object. This means that participants had to resist interference from their knowledge of the true location of the object. Second, participants’ task was different as this time they were asked to infer which of the two boxes the woman would open first to find the green object. Therefore, in the false-belief trials, the correct answer required inferring that the woman will open the box where the object was not located (because she did not see that the boxes had been swapped).

False-belief trials (*n* = 12) were mixed with true-belief trials (*n* = 12), memory control and antistrategy filler trials (*n* = 12). All trials (*n* = 36) were presented in two different blocks during the same session.

In this version of the false-belief task, the demands in spontaneous belief tracking were lower than in the reality unknown version described earlier because the question (‘Which of the two boxes will the woman open first?’) invited participants to take into account the woman’s perspective by directing their attention to the woman’s actions. The demands in self-perspective inhibition, on the other hand, were higher because participants had to inhibit their own knowledge of the true location of the object (because this time they saw where the object was truly located). Similarly to the previous task, the demands in terms of inhibition of irrelevant distractors were low since no irrelevant information was provided in the scenarios.

#### Task 3: reality unknown false-belief task with distractors

This task was an adaptation of the Nosy Neighbor Task (Samson *et al.*, 2007) and consisted of non-verbal short videos inserted within a PowerPoint presentation. It was explained to the participants that the videos placed within the window of a house allowed them to see what the woman inside the house was doing with boxes and objects. Different characters, called neighbors, passed in front of the window. It was further explained that when a neighbor stopped in front of the window, he could see what happened inside the house. But when the neighbor did not stop and continued to walk, he could not see what happened inside the house.

In each video, a woman manipulated different objects and boxes. She used scarves on top of the boxes to hide the manipulated objects so that the participant could not see the objects (this is why this task is also called here ‘reality unknown’). After putting an object in the box, the woman showed the content of the box to the neighbor when he was present in front of the window. At the end of each video, the participant’s task was to tell which object was inside the box. Two response options were proposed. The neighbor’s role was to help the participant to find the correct content of the box. For that, he indicated with his finger one of two contents proposed after each video.

In this task, the false-beliefs trials (*n* = 12) were mixed with true-belief trials (*n* = 12). In the false-belief trials, the neighbor saw the first object placed in the box but then walked away so that he could not see that the woman changed the content of the box. In contrast, for the true-belief trials, the neighbor stayed always at the window during all the video.

Crucially, the boxes were familiar containers in which a specific content could be expected. The false and true-beliefs trials consisted each of ‘filler’ trials (*n* = 4) and ‘target’ trials (*n* = 8). In the filler trials, the correct response corresponded to the salient and predictable content (that is the content that was the most likely content for the container, e.g. a chocolate in a chocolate box) whereas in the target trials, the correct response corresponded to an object not usually placed in the container (e.g. a candle in a chocolate box). The comparison between the filler and target trials allowed us to detect if participants had difficulties in inhibiting the irrelevant distractors to infer the other’s perspective. Thus, if a participant has a deficit in inhibiting irrelevant distractors, he should have an impaired performance for the target trials but a good performance for the filler trials irrespective as to whether it was a true- or a false-belief trial. All 24 trials were presented in two different blocks within the same session.

In this version of the false-belief task, the demands in terms of spontaneous belief tracking were high. As for the reality unknown false-belief task without distractors (task 1), the test question (‘What is in the box?’) did not refer to the neighbor’s mental state. Yet, to successfully respond to the task, participants had to take the neighbor’s belief into account. The demands in terms of self-perspective inhibition were low for the same reason as for task 1 (the manipulated objects in the video were hidden by scarves and thus participants did not know which content was inside the container before they inferred that the neighbor had a false belief). Finally, the demands in terms of inhibition of the irrelevant distractors were high because for the targets trials, participants had to be able to inhibit the irrelevant and predictable content (e.g. a chocolate in a chocolate box) to choose the correct answer.

#### Task 4: reality known false-belief task with distractors

This task was also an adaptation of the Nosy Neighbor Task (Samson *et al.*, 2007) and consisted of similar non-verbal short videos as task 3. However, in this task, the participants could see the manipulated contents in all the videos (the woman did not use scarves to hide the objects), and the test question was ‘What does she/he (the neighbor) think there is in the box?’. The same familiar containers and contents as those used in task 3 were used here.

False-belief trials (*n* = 12) were mixed with true-belief trials (*n* = 12). Each belief trials consisted of target trials (*n* = 8) and filler trials (*n* = 4). All trials (*n* = 24) were presented in two different blocks during the same session.

In this version of the false-belief task, the demands in terms of spontaneous belief tracking were lower because the test question [‘What does she/he (the neighbor) think there is in the box?’] invited explicitly participants to take into account the neighbor’s perspective by directing participants’ attention to the neighbors’ thoughts. The demands in self-perspective inhibition were higher than in the reality unknown with distractors task (task 3) because participants had to inhibit their own knowledge about the last object placed inside the container to infer the neighbor’s belief. Finally, the demands in terms of inhibition of irrelevant distractor were high for the same reason as for task 3.

All patients and controls were presented the tasks in the same order, starting with task 1, followed by task 2, then task 3 and finally task 4.

The research protocol was approved by the biomedical ethic committee of the Cliniques universitaires Saint-Luc (registration numbers B403201316188 and B403201112043). All the participants gave their written consent and received financial compensation for their participation.

## Results

### Neuroanatomical analysis

**Fig. 1.**
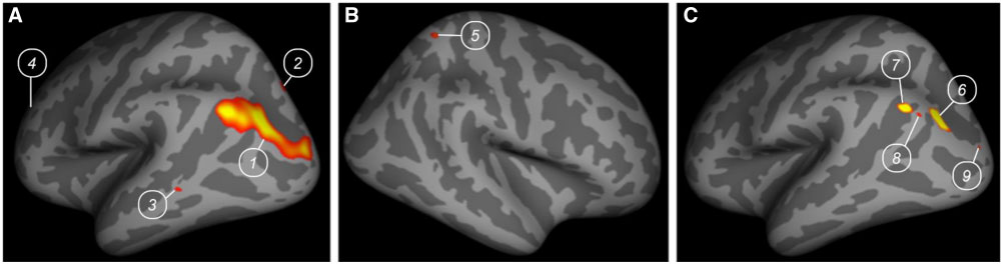
Regions showing significantly reduced cortical thickness in the two patients compared with 18 healthy controls (A) in the left hemisphere, (B) in the right hemisphere and (C) in the left hemisphere when the biggest cluster is decomposed into main activation peaks. Numbers are used for cross-reference with Table 1.

Surface-based analyses of MRI data showed that the strongest common reduced cortical thickness across both patients was in the left inferior parietal area (Figure 1 and Table 1), a region corresponding to the left posterior TPJ (TPJp) as defined according to recent anatomical parcellation studies of the TPJ (Mars *et al.*, 2012; Igelstrom *et al.*, 2015). This region was also demonstrated to be the region for which the cortical thickness was significantly different for each patient separately compared with their matched control group (Table 2).

**Table 1:** List of the brain regions for which the cortical thickness was significantly different in patients KV and IM compared with the control group (*n* = 18)

			TAL coordinates			
Anatomical region	Cluster *N*°	Size (mm^2^)	*X*	*Y*	*Z*	Max ( = −10log *p*)
In the left hemisphere (Figure 1A):						
Inferior parietal	1	1608.49	−34.2	−81.7	28.5	5.8863
Superior parietal	2	19.82	−19.2	−79.5	42.4	3.4085
Bank of superior temporal sulcus	3	11.49	−60.4	−48.9	0.5	3.2782
Rostral middle frontal	4	2.26	−26.2	46.2	14.5	3.1417
In the right hemisphere (Figure 1B):						
Superior parietal	5	25.38	27.7	−54.3	63.6	4.3835
In the left hemisphere: decomposition of the cluster N°1 in main activation peaks (Figure 1C):						
Inferior parietal	6	149.08	−34.2	−81.7	28.5	5.8863
Inferior parietal	7	68.85	−39.9	−67.5	33.6	5.7026
Inferior parietal	8	8.38	−42.4	−73.8	31.6	5.0745
Lateral parietal	9	4.62	−22.3	−94.7	15.3	5.0741

The coordinates are in the Talairach space (TAL).

**Table 2:** List of the brain regions for which the cortical thickness was significantly different for each patient separately compared with their matched control group (*n* = 10 for patient IM, *n* = 8 for patient KV)

			TAL coordinates			
Anatomical region	Cluster *N*°	Size (mm^2^)	*X*	*Y*	*Z*	Max ( = −10log *p*)
In the left hemisphere:						
For patient IM:						
Inferior parietal	1	1356.04	−35.0	−81.9	18.1	4.2507
For patient KV:						
Superior parietal	1	1027.31	−11.2	−73.4	49.0	5.6829

The coordinates are in the TAL. Note that for the right hemisphere, when both patients were compared separately with their control group, no region showed a significant reduction in cortical thickness.

### Behavioral tasks

**Fig. 2.**
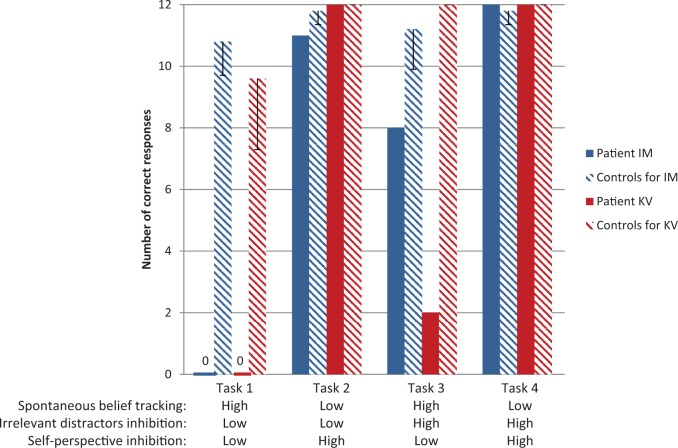
Upper part: total number of correct responses for false-belief trials (maximum = 12) for the two patients and mean number of correct responses for the matched controls (5 controls for each patient) in the four experimental tasks. The error bars represent s.d.. Lower part: summary of the mentalizing demands in each task.

In all tasks, the two patients showed a good performance for the control trials. All scores were either not significantly different from the scores of the control subjects (Crawford and Howell, 1998s modified *t*-test, *t*(4) < −1.623, *P*(one-tailed) < 0.089) or, when the variability in the controls’ scores was nil and statistical tests could not be applied, the patients’ scores were above the chance level cutoff of 9/12 correct (Figure 2 and Supplementary Table S2).

The patients’ profile of performance showed a marked and common deficit in belief reasoning in only two out of the four tasks: the only two tasks which placed high demands in spontaneous belief tracking. In one of these tasks (task 1), the two patients did not get a single correct response on the false-belief trials, showing a clear impairment compared with controls (Crawford and Howell, 1998s modified *t*-test; patient IM: *t*(4) = −8.963, *P*(one-tailed) < 0.001; patient KV: *t*(4)= −3.810, *P*(one-tailed) < 0.01). In the other task (task 3), the patients’ performance was also poor and significantly lower compared with the score of the controls (Crawford and Howell, 1998s modified *t*-test; patient IM: *t*(4) = −2.247, *P*(one-tailed)* *= 0.04; patient KV: no statistics could be performed but the ceiling effect observed in the controls markedly contrasted with the score of 2/12 achieved by the patient).

In contrast, the two patients could correctly infer someone else’s false belief despite high demands in self-perspective inhibition (task 2) or high demands in salient distractor inhibition (task 4). In task 2, patient IM scored 11/12 (a score not significantly different from the score of the controls, Crawford and Howell, 1998s modified *t*-test; *t*(4) = −1.623, *P*(one-tailed) = 0.09) and patient KV scored at ceiling like the controls. In task 4, both patients scored at ceiling.

## Discussion

Overall, the results across the four false-belief reasoning tasks demonstrated that both patients can be classified as exhibiting a classical dissociation with a marked difference of performance between the tasks in which there were no explicit mentalizing instructions (impaired performance on tasks 1 and 3) and the tasks where attention was explicitly directed to the agent’s thoughts or actions (spared performance on tasks 2 and 4; Supplementary Table S3). It could be argued that such a classic dissociation only results from the differential level of difficulty of the tasks in which the patients were impaired (tasks 1 and 3) compared with the tasks in which the patients showed no deficit (tasks 2 and 4). Although it is true that the performance of the controls was slightly better in tasks 2 and 4 compared with tasks 1 and 3, none of the controls showed such a floor performance as the one shown by the patients. Tasks 1 and 2 have been recently presented to over 100 healthy participants aged between 18 and 74 and none of the healthy controls showed a performance as poor as the one exhibited by patients IM and KV (B,A., M,G., A,I.A., & S,D. in preparation). Furthermore, the reverse dissociation to the one documented here has been observed in another patient following a lesion to the right lateral prefrontal cortex (Samson *et al.*, 2005). For these two reasons, a mere sensitivity to task difficulty is not sufficient to explain the extreme dissociation observed in patients IM and KV.

Besides the varying need for spontaneous belief tracking, there is another feature that distinguishes tasks 1 and 3 from tasks 2 and 4, namely the type of false belief content that can be attributed while the scenarios unfold. Indeed, in both tasks in which the patients were impaired and at the time they could infer that the woman has a false belief, they themselves did not know the location of the object (task 1) or the identity of the object placed in the container (task 3). Thus, they could only attribute a false belief (or ignorance) about the fact that the containers were tampered with but they could not attribute a belief about the object location or object identity. In contrast, in both tasks in which the patients were unimpaired, the initial location and/or initial identity of the object was revealed during the change of location (task 2) or the change of identity (task 4), so that this information could be used straightaway to attribute the false-belief content. This difference is important to take into account. Indeed, it has been recently suggested that humans have mechanisms which help track beliefs automatically (Apperly and Butterfill, 2009; Kovács, 2015; Schneider *et al.*, 2015). However, such mechanisms have been found to have limits, such as not being able to deal with complex object identities (Low and Watts, 2013) or with absent objects (Kovács *et al.*, 2014). Whether such mechanisms can track an unknown object location or an unknown object identity is still not clear (for a discussion see Kovács, 2015). In the case of our patients, this could mean two things. First, the putative low-level mechanisms for tracking automatically beliefs may be impaired and such impairment may be directly at the origin of the patients’ difficulties. Or alternatively, the patients’ low-level mechanisms may still be operational but could not help them as the situation required operations beyond their processing capacity. In that latter case, the origin of the patients’ impairment would be unrelated to the putative low-level belief tracking mechanisms. Either way, our data show that the patients had difficulties inferring spontaneously the other person’s belief. Their deficit may however be limited to cases where the belief relates to an unknown object location or an unknown object identity.

Interestingly, directing directly the patients’ attention to the other person’s thoughts (task 4) and only directing attention to the other person’s action (task 2) were sufficient to trigger accurate mentalizing in both patients. In contrast, simply informing at the beginning of the tasks that the other person may help them find out an object location (task 1) or an object identity (task 3) did not trigger accurate mentalizing. In those latter cases, the repeated instructions on every trial to find the object location and the object identity (without explicit reminder that the other person will help) may have diverted attention away from the other person. One interpretation could be that the processes impaired in both patients are processes which usually help healthy participants realize that the other person may have a false belief (perhaps something like an alarm signal triggered in a bottom–up fashion) which then attracts more sustained attention to the other person and which in turn triggers explicit mentalizing. In tasks 2 and 4, where every test questions directly relates to the other person and hence attracts attention to the other person in a top–down fashion, the bottom–up alarm signal may not be necessary anymore to trigger explicit mentalizing. This remains still speculative and more research is needed to better understand the interplay between attention and mentalizing.

Given that the largest commonly lesioned brain area in both patients was the left TPJp, the evidence reported here strongly suggests that this area is necessary to spontaneously infer and take into account the perspective of others in the absence of explicit instructions (at least in the cases of beliefs about unknown object location or unknown object identity). Although the left TPJp appears to be causally linked to the patients’ difficulty, this is not to say that spontaneous belief tracking is only sustained by the left TPJp. Efficient spontaneous belief tracking is probably achieved by efficient connections between the left TPJp and other brain areas involved in ToM. Further research may shed new lights on how this connectivity works. Schuwerk *et al.* (2014) recently suggested that the left TPJ may send early signals to the medial prefrontal cortex which then communicates back to the left and right TPJ. This suggestion was based on dynamic causal modeling of data obtained in a belief reasoning task in which participants were explicitly instructed to reason about beliefs (Schuwerk *et al.*, 2014). It would be interesting to follow-up this suggestion with activation data obtained in a task in which there are no explicit mentalizing instructions.

The difficulties in spontaneous belief tracking of the two patients reported here could be further interpreted in two ways. First, it is possible that the two patients did not compute at all the other person’s false belief unless they were instructed to track the person’s mental state. It has been recently claimed that the left TPJ, more particularly the left inferior parietal lobule (IPL), may play a general role in consciously tracking potential perspective differences (Arora *et al.*, 2015). The profile of our patients would suggest that the left TPJ tracks such perspective difference *spontaneously*. Second, it is possible that the two patients had no problems in computing the protagonist’s false belief but failed to *spontaneously use* the results of the computation at the time of their response. Such a problem could not be a general memory problem, since the performances of both patients on memory control trials were good. Instead, the difficulties would be in line with a recent proposal that the left TPJ may play a specific role in the automatic awareness of task-relevant information retrieval from memory (for a review see Berryhill, 2012; see also Cabeza *et al.*, 2012). These two aspects of spontaneous mental state tracking need to be further examined in future research.

Even if spontaneously the two patients did not report difficulties in their social interactions, when more specific questions were asked, both patients acknowledged more frequent occurrences of misunderstandings in their relations with others. This points to the fact that there were everyday life consequences of their deficits but that these were quite subtle.

Finally, our study supports the idea that neuropsychological single case studies are important and complementary to functional neuroimaging studies in healthy subjects as they provide important empirical evidence to understand the functional role of the various brain areas part of the ToM network. One of the early examples of such influence is the report by Bird *et al.* (2004) of a patient with an extensive frontal lobe damage encompassing the mPFC but who was unimpaired in ToM tasks thereby strongly constraining interpretations of the role of the mPFC in ToM (for a discussion see, Isoda and Noritake 2013).

To summarize, successful navigation in the social world requires spontaneously detecting other people’s discrepant perspectives, and retrieving this information when it becomes relevant for interpersonal interaction. The two cases of patients reported here allowed us to identify one of the brain areas that plays a necessary role to achieve this, namely the left TPJp. Although several studies have shown that the right TPJ plays a more specific role in mental state attribution (people’s beliefs, intentions and emotions) than the left TPJ (Saxe and Powell, 2006; Aichhorn *et al.*, 2009), we bring new evidence showing that the role of the left TPJ is not less important for efficient ToM processing.

## Supplementary Material

Supplementary Data
