# Supplementary material for: Impaired spontaneous belief inference following acquired damage to the left posterior temporoparietal junction
Source: Soc Cogn Affect Neurosci. 2016 Jun 17;11(10):1513–20. doi: 10.1093/scan/nsw076 (PMC5040917; doi:10.1093/scan/nsw076)
Supplement: Supplementary Data [file supp_11_10_1513__index.html]

Impaired spontaneous belief inference following acquired damage to the left posterior temporoparietal junction — Supplementary Data 

# Impaired spontaneous belief inference following acquired damage to the left posterior temporoparietal junction

## Supplementary Data

files

- Supplementary Data - pdf file
