# Supplementary material for: Impaired spontaneous belief inference following acquired damage to the left posterior temporoparietal junction
Source: Soc Cogn Affect Neurosci. 2016 Jun 17;11(10):1513–20. doi: 10.1093/scan/nsw076 (PMC5040917; doi:10.1093/scan/nsw076)
Supplement: Supplementary Data [file supp_nsw076_scan-16-213-File006.pdf]

Table supplement 1. Neuropsychological profile of the patients IM and KV.

Test	Patient IM		Patient KV		
	2011	2012-2013	2010	2012	2014
Working Memory					
Storage					
Arithmetic Span[a] (forwards)	7	-	**3**	4	4
Spatial Span[a] (forwards) (NS)[b]	9	-	**4**	**2**	**3**
Manipulation					
Arithmetic Span (backwards)	5	-	3	2	2
Spatial Span (backwards) (NS)[b]	10	-	7	**4**	6
Long-term Memory					
Visual					
Doors Test[c] – Part A	12/12	-	11/12	11/12	9/12
– Part B	11/12	-	8/12	8/12	9/12
– Total	23/24	-	19/24	19/24	18/24
Verbal					
Buschke 16 items[d]					
Immediat recall	16/16	16/16	16/16	**-**	16/16
Recall 1 – Free	**4/16**	11/16	5/16	**-**	7/16
– Total	16/16	16/16	9/16	**-**	**11/16**
Recall 2 – Free	**5/16**	15/16	**3/16**	**-**	**5/16**
– Total	15/16	16/16	**8/16**	**-**	**10/16**
Recall 3 – Free	**5/16**	14/16	**4/16**	**-**	**8/16**
– Total	15/16	16/16	**6/16**	**-**	15/16
Recognition	16/16	16/16	**6/16**	**-**	**14/16**
Delayed free recall – Free	**8/16**	15/16	**1/16**	**-**	**8/16**
– Total	15/16	16/16	**5/16**	**-**	15/16
Executive Functions					
Stroop Test[e]					
Colors Naming – Time (s)	**76**	61	70	**87**	**96**
– uncorrected Errors	0	0	1	**4**	0
Words Reading – Time (s)	**53**	44	55	63	**83**
– uncorrected Errors	0	0	0	**3**	0
Inhibition – Time (s)	**236**	101	**175**	**211**	178
– uncorrected Errors	**5**	0	0	1	**4**
Inhibition-Naming – Time (s)	**160**	40	**105**	**124**	82
– uncorrected Errors	**5**	0	-1	-3	**4**
Trail Making Test[f]					

Part A – Time (s)	22	23	73	60	77
– Errors	0	0	0	0	0
Part B – Time (s)	69	78	**343**	**311**	<u>225</u>
– Errors	0	0	**4**	0	1
Brixton Test[g]					
Errors	-	-	**-**	-	5
Fluency					
Category fluency (animals)	29	-	**16**	<u>21</u>	28
Letter fluency (P)	15	18	<u>13</u>	22	21
Attention					
D2 cancellation task[h]					
Speed	**234**	**330**	-	**-**	402
Quality	0,8%	1,2%	-	**-**	10,44%
Profitability	**232**	<u>326</u>	-	**-**	360
Regularity	8	9	-	**-**	23
Logical Reasoning					
Progressives Matrices (PM38)[i]	52/60	-	<u>29/60</u>	**26/60**	36/60

The impaired performances (the cut-off score -2 *SD* or <P5, is based on published norms for each test) are **highlighted in bold**. The borderline performances (between -1.4 *SD* and 1.9 *SD* or between P9 and P3) are <u>underlined</u>.

[a] Wechsler (1997).
[b] N.S = standard note
[c] Baddeley, Emslie & Nimmo-Smith (1994)
[d] Epreuve de Rappel Libre/Rappel Indicé à 16 items – Van der Linden et al. (2004)
[e] Stroop (1935).
[f] Reitan & Wolfson (1985)
[g] Burgess & Shallice (1997)
[h] Brickenkamp (1981)
[i] Raven, Raven & Court (1998)

Table supplement 2. Patients IM's and KV's performance (and mean of scores and standard deviation of their matched control subjects) across the four tasks and the different types of trials. We first compared the patients' number of correct responses for all trials in each of the four tasks to the number of correct responses of their matched control subjects by means of a modified t-test which allows comparing one person's performance to that of a group of controls (Crawford and Howell, 1998). When the variability in the controls' score was nil, the modified t-test could not be applied. In those cases, we considered the score of 10/12 as the limit for a spared performance for the following reasons. Firstly, one or two errors may be caused by distraction. Secondly, the score of 10/12 was statistically significantly different from chance level (one-tailed P-value associated with getting 10/12 correct = 0.019 by binomial test) and above the chance level cutoff of 9/12. **Impaired scores are in bold.**

	Patient IM (Mean of controls ± *SD*)	IM compared to controls (modified *t*-test, one-tailed *p*)	Patient KV (Mean of controls ± *SD*)	KV compared to controls (modified *t*-test, one-tailed *p*)
**Task 1**				
FB (n=12)	**0** (10.8± 1.10)	**t(4) = -8.963, p = 0.000**	**0** (9.6± 2.30)	**t(4)= -3.810, p = 0.009**
TB (n=12)	12 (12± 0.00)	n/a	12 (11.6± 0.89)	t(4)= 0.410, p = 0.351
MC (n=12)	12 (11.6± 0.55)	t(4) = 0.664, p = 0.271	11 (12± 0.00)	n/a
IC (n=6)	6 (6± 0.00)	n/a	6 (6± 0.00)	n/a
Fillers (n=6)	6 (6± 0.00)	n/a	6 (6± 0.00)	n/a
**Task 2**				
FB (n=12)	11 (11.8± 0.45)	t(4) = -1.623 , p = 0.089	12 (12± 0.00)	n/a
TB (n=12)	12 (11.6± 0.89)	t(4)= 0.410, p = 0.351	10 (12± 0.00)	n/a
MC + fillers (n=12)	12 (12± 0.00)	n/a	11 (12± 0.00)	n/a
**Task 3**				
FB (n=12)	**8** (11.2± 1.30)	**t(4) = -2.247, p = 0.044**	**2** (12± 0.00)	n/a
TB (n=12)	12 (12± 0.00)	n/a	11 (11.8± 0.45)	t(4) = -1.623 , p = 0.089
**Task 4**				
FB (n=12)	12 (11.8± 0.45)	t(4)= 0.406, p = 0.352	12 (12± 0.00)	n/a
TB (n=12)	12 (12± 0.00)	n/a	11 (12± 0.00)	n/a

FB = False belief; TB = True belief; MC = Memory control; IC = Inhibition control; n/a = not-applicable.

Table supplement 3. In a single case design, a classical dissociation is established (1) when the patient has a deficit on Task X and is within normal limits on Task Y, and (2) when there is a significant difference between performances on Task X and Y (Crawford, Garthwaite, & Gray, 2003). Thus, in an additional set of analyses, we compared the patients' performance on the false belief trials across tasks 1 and 2 on the one hand and, across tasks 3 and 4 on the other hand by means of the Revised Standardized Difference Test (Crawford and Garthwaite, 2005).

	Comparison between task 1 and task 2	Comparison between task 3 and task 4
Patient IM	$t(4)$= 4,981, $p_{\text{two-tailed}}$ = 0,007	$t(4)$= 4,522, $p_{\text{two-tailed}}$ = 0,0106
Patient KV	n/a<br>but the difference between the two tasks was very large (0/12 and 12/12) and the patient's performance in task 1 was below the chance level cutoff.	n/a<br>but the difference between the two tasks was quite large (8/12 and 12/12) and the patient's performance in task 3 was below the chance level cutoff.
